# Dyspnea Index: An upper airway obstruction instrument; translation and validation in Swedish

**DOI:** 10.1111/coa.13682

**Published:** 2021-01-03

**Authors:** Eleftherios Ntouniadakis, Ole Brus, Mathias von Beckerath

**Affiliations:** ^1^ Department of Ear Nose and Throat Faculty of Medicine and Health Örebro University Örebro Sweden; ^2^ Clinical Epidemiology and Biostatistics Faculty of Medicine and Health Örebro University Örebro Sweden

**Keywords:** Dyspnea Index, laryngotracheal stenosis, paradoxical vocal fold movement, Patient‐Reported Outcome Measures, quality of life, upper airway obstruction, validation

## Abstract

**Objective:**

Upper airway dyspnoea is a challenging condition in which assessing the discomfort experienced by the patient is essential. There are three patient‐reported outcome (PRO) instruments developed particularly for this patient group, none of which is available in Swedish. The aim of this study was to translate the Dyspnea Index (DI) into Swedish and validate the instrument for use in the Swedish‐speaking population by investigating its basic psychometric properties.

**Design:**

A prospective instrument validation study.

**Setting:**

Tertiary referral centre.

**Participants:**

Fifty‐three (n = 53) patients with upper airway dyspnoea and 19 healthy controls.

**Main outcome measures:**

The questionnaire was translated into Swedish (swDI) with a forward‐backward method. Reliability, repeatability, responsiveness and construct validity were assessed by asking the subjects to complete the swDI, a visual analog scale (VAS) at exertion and at rest and the Voice Handicap Index (VHI).

**Results:**

The swDI showed excellent internal consistency (Cronbach's α: 0.85) and repeatability (interclass correlation coefficient: 0.87 and Pearson's *r*: .89) in the patient group. No ceiling effect was observed (maximum score achieved was 39; 85% of the patients scored ≤ 36). SwDI scores moderately correlated with VAS at exertion (*r*: .59) and at rest (*r*: .42), yet poorly with the VHI (*r*: .36). The effect size (ES) was 3.8.

**Conclusions:**

The swDI is a valid, robust and reliable questionnaire for self‐assessment in Swedish‐speaking patients with upper airway obstruction. A future anchor‐based longitudinal study is needed to assess the smallest detectable change (SDC) and minimal important change (MIC) that were not estimated in our study.

**Key points**

- Dyspnea Index (DI) the only available instrument developed exclusively for the assessment of upper airway dyspnea is now translated in Swedish
- The Swedish version of Dyspnea Index (swDI) is a valid instrument showing good psychometric properties
- SwDI's psychometric properties are in agreement with the COnsensus‐based Standards for the selection of the health Measurement INstruments (COSMIN) initiative guidelines
- SwDI is now available to be used both in clinical practice and research

## 1 INTRODUCTION

Upper airway dyspnoea is difficult to evaluate by means of any functional examination without considering the severity of the symptoms reported directly by the patient. The extent of discomfort may affect the type and priority of the intervention both in acute and recurring conditions. There are several ways to assess dyspnoea clinically (eg flexible video laryngoscopy and computed tomography) as well as functionally (spirometry).^1^, ^2^, ^3^

The severity of the symptoms due to obstruction of the upper airway may vary. A structural obstructive lesion (eg laryngotracheal stenosis, tumours, and vocal fold ankylosis) normally presents with constant breathing problems at exertion but also at rest in severe cases.^1^, ^2^ Functional or other non‐structural disorders (eg paradoxical vocal fold movement (PVFM) and laryngospasm) occasionally occur in asymptomatic patients upon exposure to a possible triggering factor.^2^, ^4^ Neurological conditions may appear in a persistent (nerve paralysis or injuries) or intermittent (laryngeal dystonia) manner.^2^, ^3^ There are no distinct anatomical margins as far as the lower part of the upper airway is concerned; however, the extrathoracic part of the trachea is commonly included.^5^, ^6^, ^7^

In a systematic review, Noud et al^1^ showed that there are only three patient‐reported outcome (PRO) instruments developed to facilitate the evaluation and follow‐up of patients with upper airway obstruction: The chronic respiratory questionnaire (CRQ) is a widely used measure of health‐related quality of life for patients with airflow limitations, targeting mostly pulmonary diseases and the lower airways.^8^ The Medical Research Council (MRC) Dyspnea Scale as well as clinical chronic obstructive pulmonary disease questionnaire (CCOPDQ) were both initially developed for lung disorders and not for the upper airways; nevertheless, they are validated for only one specific disease in the upper airway (adult laryngotracheal stenosis).^9^, ^10^ The Dyspnea Index (DI) was found to be the only validated questionnaire uniquely developed for and used in various types of upper airway dyspnoea.^1^, ^4^, ^11^, ^12^ Considering that none of the aforementioned instruments is available in Swedish, we were prompted to translate and validate the DI.

The DI is a questionnaire initially developed by Gartner‐Schmidt et al to quantify the severity of symptoms, particularly in adults with upper airway dyspnoea,^4^ which was later validated for adolescents with PVFM.^11^ The original English version of DI, shown in Appendix 1, is a Likert scale instrument including 10 items. The respondent is asked to evaluate the frequency of each statement using a 5‐point interval scale (0 = never, 1 = almost never, 2 = sometimes, 3 = almost always, 4 = always). The total score ranges from 0 to 40. A higher cumulative sum score is associated with more severe dyspnoea.^4^

The purpose of the present study was to translate the DI into Swedish and validate the instrument for use in the Swedish‐speaking population by investigating its basic psychometric properties such as reliability in terms of internal consistency, reproducibility, construct validity and responsiveness.

## 2 MATERIALS AND METHODS

### 2.1 Ethical considerations

This study was performed in accordance with the Declaration of Helsinki. This human study was approved by Ethics Review Board in Uppsala. All parents, guardians or next of kin provided written informed consent for the minors to participate in this study. All adult participants provided written informed consent to participate in this study.

### 2.2 Translation

The English version of the DI was translated into Swedish by using the forward‐backward method as described below. An authorised translator and two experienced otorhinolaryngologists, native Swedish speakers fluent in English, independently translated the questionnaire to Swedish. The reconciliation of the forward translations on a common pilot version was made by the first and the last author of this manuscript. This pilot version was then translated back to English by another authorised translator who did not participate in the initial forward translation and was not aware of the original version. The back translation was then compared with the original DI. For those items for which the back–translated and the original DI did not match, the choice of words was discussed by the aforementioned authors until an agreement was reached and a final version was reconciled. A pilot group of physicians with various backgrounds and experience was created. The group answered the questionnaire after the backward translation was applied, and their comments were taken into consideration to produce the final Swedish version of the Dyspnea Index (swDI), as seen in Appendix 2.

### 2.3 Validation

All patients with upper airway obstruction were recruited at the Ear Nose and Throat Department at Örebro University Hospital, Sweden, between January 2017 and July 2019. The diagnosis was made by otorhinolaryngologists with experience in airway problems after thorough history taking and a physical examination, including flexible video laryngoscopy. The swDI was then administered to the patients as soon as they agreed to participate in the study. Due to the fact that upper airway dyspnoea is an uncommon condition, a sample size calculation based on a power analysis was not performed. Thus, our goal was to recruit 50 patients to reach a sample size equivalent to preceding DI validation studies,^4^, ^11^ estimating that this could be achieved with data collected within 2 years. The control group, all of them oncology and otorhinolaryngology residents, were recruited while participating in a Head and Neck Cancer training course held at our clinic in November 2017. None of them were involved in the translation process.

All patients who were planned to undergo an intervention were asked to complete the Swedish version of the Voice Handicap Index (VHI) before and after treatment. The VHI is a robust, extensively used, validated instrument in Swedish for the self‐assessment of voice problems.^13^ Our intention was to investigate whether VHI score correlates to DI score since phonation and breathing are two different functions diversely affected by conditions in this airway subsite. Behavioural therapy was offered exclusively to those diagnosed with PVFM by speech‐language pathologists with special expertise in this particular condition. The remaining patients were treated surgically with conventional endoscopic procedures under general anaesthesia (CO_2_ laser cordectomy, balloon dilatation, excision with cold instruments) by experienced airway surgeons in our department.

Subjects who were incapable of making informed, intelligent and voluntary decisions, under the age of 14, or non‐fluent Swedish speakers were excluded from the study.

Our intention was to evaluate swDI psychometric properties in accordance with the COnsensus‐based Standards for the selection of the health Measurement INstruments (COSMIN) initiative guidelines.^14^

### 2.4 Reliability

The reliability of the swDI was assessed with Cronbach's reliability coefficient α^15^ calculating the interitem internal consistency among the patient group before and after intervention, the control group separately and all study subjects. Eventual floor or ceiling effects were examined. The interclass correlation coefficient (ICC) with individual modelled as a random effect, Pearson's and Spearman's correlation coefficient (r)^15^ were used to evaluate the agreement between two repeated measurements facilitating comparisons between the present study and other existing validations^4^, ^11^ using the same method.

### 2.5 Construct validity

Known‐group validation was performed by means of an independent samples t test and Mann‐Whitney test between the patient group and the control group. Additionally, the subjects in the patient group were asked to subjectively rate their breathing problems at rest and at exertion on a visual analog scale (VAS) in parallel with completing the swDI. The VAS was a 100‐mm long, straight, horizontal line where 0 mm represented “no breathing problems at all” and 100 mm represented “worst possible breathing problem.” Furthermore, all subjects receiving a treatment were asked to fill in the Swedish version of the VHI. Subsequently, correlations between the swDI and VAS and the swDI and VHI were calculated with Pearson's and Spearman's correlation coefficient in an attempt to evaluate swDI’s convergent and discriminant validity, respectively.^15^

### 2.6 Responsiveness

The patient group was requested to fill in the questionnaire before and approximately 2 months following a surgical intervention, or a completed speech‐behavioural therapy carried out by a speech therapist depending on the diagnosis. A paired *t* test calculating the effect sizes (ES) and a Wilcoxon signed‐ranks test was then performed to estimate swDI responsiveness.

### 2.7 Statistics

**FIGURE 1:**
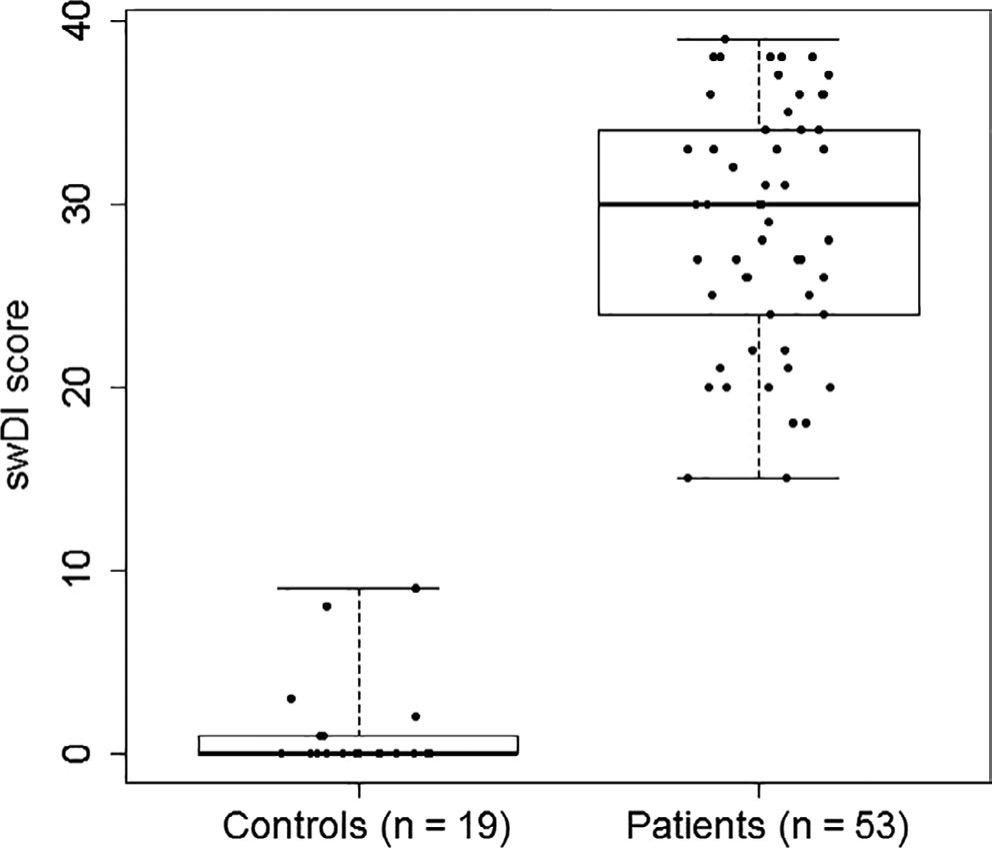
Boxplot with individual values (dots) showing swDI scores in the patient and the control group. The line across the box indicates the median. Outliers are cases with values between 1.5 and 3 times the IQ range, that is beyond the whiskers. Extremes are cases with values more than three times the IQ range. Patients with upper airway obstruction score significantly higher in the swDI compared to the controls (*P* < .001)

IBM® SPSS® Statistics version 25 was used for the statistical analysis and Figure 1was created in R version 3.6.0.240613.

## 3 RESULTS

The study population consisted of 72 subjects (Table 1), including 53 consecutive patients diagnosed with breathing problems due to conditions of the upper airway, and 19 healthy controls. Forty‐three (n = 43) of the patients received an intervention (surgical or speech‐behavioural). The various diagnoses set in the patient group are shown in Table 2. The subjects involved in each part of the validation process are shown in Table 3.

**TABLE 1:** Demographic characteristics of the study group

	Median age (min–max)	Females	Males
Patients	55.5 (14.2‐81.8)	45	8
Controls	36.6 (32.3‐50.6)	10	9
Total	50.5 (14.2‐81.8)	55	17

**TABLE 2:** Diagnosis set in the patient group

	Number of patients
Laryngotracheal stenosis	41
Paradoxical Vocal Fold Movement	5
Bilateral Vocal Fold Ankylosis	5
Reinke's oedema	2

**TABLE 3:** Subjects involved in each part of the validation process

	Patient group	Control group
Internal consistency	53	19
Test‐retest	25	19
Known‐groups validity	53	19
Convergent validity	52	0
Divergent validity	43	0
Responsiveness	43	0

### 3.1 Reliability

The analysis of internal consistency showed a Cronbach's α of 0.85 in the patient group. The maximum score achieved was 39, whereas 85% of the patients scored ≤ 36; thus, no ceiling effect was observed. When evaluating repeatability, the ICC was 0.87 (95% CI: 0.74‐0.94, *P* < .001), Pearson's correlation coefficient was 0.89 (*P* < .001) and Spearman's ρ was 0.90 (*P* < .001) in the patient group. The same calculations when made in the whole subject group (patients and controls) revealed a Cronbach's α of 0.97, ICC of 0.98 (95% CI: 0.97‐0.99, *P* < .001), Pearson's coefficient of 0.98 (*P* < .001) and Spearman's ρ of 0.95 (*P* < .001).

### 3.2 Construct validity

The 53 participants from the patient group (Mean: 28.9, SD: 6.7, SEM: 0.9) scored significantly higher swDI score compared with the 19 controls (Mean: 1.3, SD: 2.7, SEM: 0.6), t(70) = −17.4, *P* < .001. The mean difference was 27.6 (95% CI: 24.5‐30.8, *P* < .001) (Figure 1), demonstrated also in the Mann‐Whitney test (*P* < .001).

The Pearson's *r* and Spearman's ρ for the swDI‐VAS at exertion were 0.59 (*P* < .001) and 0.63, respectively. Between swDI and VAS at rest Pearson's *r* shown to be .42 (*P* = .002) and Spearman's ρ 0.42 (*P* = .002). When swDI was compared to the VHI, the Pearson's *r* and Spearman's ρ were found to be 0.36 (*P* = .018) and 0.29 (*P* = .059) each.

### 3.3 Responsiveness

**FIGURE 2:**
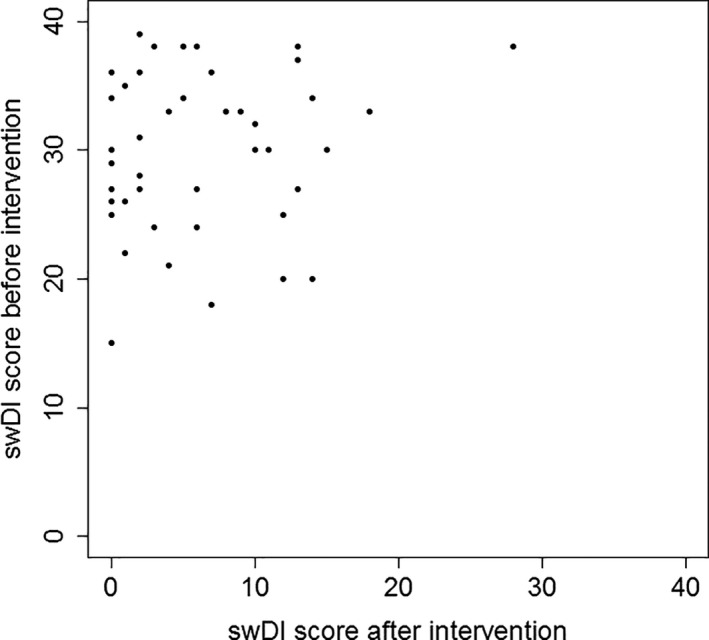
Scatterplot showing swDI scores before and after intervention (n = 43, mean difference = 23.8, *P* < .001)

A significant decrease was found when swDI scores before intervention (Mean: 30.0, SD: 6.2, SEM: 1.0) were compared with after intervention (Mean: 6.3, SD: 6.2, SEM: 1.0), t(42) = 19.2, *P* < .001. The ES was then calculated to be 3.8 (Figure 2). The related‐samples Wilcoxon signed‐ranks test showed a statistically significant change in swDI after intervention (Z = −5,714, *P* < .001).

A summary of the swDI’s psychometric properties and the recommended cut‐off values according to the COSMIN taxonomy^14^ is shown in Table 4.

**TABLE 4:** Summary of the psychometric properties of the Swedish version of the Dyspnea Index and criteria for good measurement properties according to Prinsen et al^14^ (CI: confidence interval)

		Swedish version of Dyspnea Index	Good measurement properties ‐ COSMIN taxonomy
Reliability	Internal consistency	*Patient group*: Cronbach's α 0.85 *Whole subject group*: Cronbach's α 0.97	Cronbach's α > 0.70
	Repeatability	*Patient group*: ICC^a^, 0.87 (95% CI: 0.74‐0.94, *P* < .001); Pearson's *r* .89 (*P* < .001), Spearman's ρ 0.90 (*P* < .001) *Whole subject group*: ICC^a^, 0.98 (95% CI: 0.97‐0.99, *P* < .001); Pearson's *r*, .98 (*P* < .001), Spearman's ρ 0.95 (*P* < .001)	ICC^a^ ≥ 0.70
	Ceiling effects	None	
	Known‐groups validity	Mean difference (independent *t* test): 27.6 (CI 24.5‐30.8, *P* < .001) Mann‐Whitney test: *P* < .001	Meaningful changes between relevant (sub)groups
Construct validity	Convergent validity	Pearson's *r* swDI^b^–VAS^c^ at exertion: 0.59 (*P* < .001) Spearman's ρ swDI^b^–VAS^c^ at exertion: 0.63 (*P* < .001) Pearson's *r* swDI^b^–VAS^c^ at rest: 0.42 (*P* = .002) Spearman's ρ swDI^b^–VAS^c^ at rest: 0.42 (*P* = .002)	Correlation with gold standard ≥ 0.70 OR Correlation with instruments measuring similar constructs ≥ 0.50
	Discriminant validity	Pearson's *r* swDI^b^–VHI^d^: 0.36 (*P* = .018) Spearman's *r* swDI^b^–VHI^d^: 0.29 (*P* = .059)	Correlation with instruments measuring dissimilar constructs 0.30‐0.50
Responsiveness		swDI before intervention: Mean: 30.0, SD: 6.2 swDI after intervention: Mean: 6.3, SD: 6.2, *P* < .001 ES^e^: 3.8 Related‐samples Wilcoxon signed‐ranks test: Z=−5,714, *P* < .001	Meaningful changes between relevant groups

^a^ Interclass correlation coefficient.

^b^ Swedish version of the Dyspnea Index.

^c^ Visual Analog Scale.

^d^ Voice Handicap Index.

^e^ Effect size.

## 4 DISCUSSION

### 4.1 Synopsis of key findings

The results of this study demonstrate that the swDI shows good psychometric properties and may be used in clinical praxis as well as in research as a complement to clinical evaluation in the assessment and follow‐up of Swedish‐speaking patients with upper airway dyspnoea. Our endeavour was to deliver a valid, robust and reliable questionnaire to be used for self‐assessment of breathing problems caused by upper airway obstruction in a Swedish‐speaking population.

### 4.2 Strengths of the study

A specific consensus regarding the process of translating a PRO instrument has not been reached.^16^, ^17^, ^18^ It is generally recommended to acquire at least 2 versions in the target language from a varied profile of forward translators, with a subsequent panel assessment to reach an agreement of a preliminary adaptation.^15^, ^17^ Considering the importance of the language used in defining the context of the translated document, we chose to engage two authorised translators ensuring a professional linguistic approach. Additionally, two experienced specialists in airway problems, the authors excluded, were consulted to maintain the intended clinical prospect in the target language. All physicians participating in this process agreed that there were no culture‐bound disease implications that would modify the context of the original version. Some items, in particular items 5 and 9 required a lengthy discussion during the translation process, as “stress” is a word with an occasionally negative meaning in Swedish apart from its nuance in American English.

However, the use of backward translation, although commonly disputed,^16^, ^19^ was used with the purpose of controlling changes altering the original meaning. Furthermore, receiving input from the target population and making appropriate modifications is considered equally important at that phase.^15^, ^16^, ^17^, ^19^, ^20^ No major comments were made from the pilot group testing of our final version of the swDI.

### 4.3 Comparisons with other studies

Subjects interpret the severity or frequency of a symptom when providing their own judgment in PROs differently. Thus, there is always the risk of classification bias.^21^ Our results suggested that the swDI is a reliable instrument in terms of internal consistency showing high Cronbach's α values. However, statement number 8 (corresponding to “my shortness of breath gets worse with exercise or physical activity”) was the only one found to poorly correlate with the other constructs in the interitem correlation matrix (data not shown). Although not significant, this finding may indicate either an inadequate function of the particular item or a classification bias as approximate response alternatives (eg almost never, sometimes and almost always) of the questionnaire could be perceived variously by different individuals.^21^

Moreover, the sample size in our study was not regarded as large enough to individually evaluate statements from the interitem correlation matrix.^15^ A larger study sample would facilitate further investigation of each component with advanced statistics such as factor analysis or item response theory.^1^, ^15^, ^22^, ^23^, ^24^ Terwee et al^23^ suggest a minimum of 100 subjects to ensure stability of the covariance matrix, whereas Cappelleri et al^24^ recommend at least 300 for a thorough individual item evaluation, pointing out that weakly correlated constructs may require more subjects for precise estimates.

Upper airway obstruction is caused by a heterogeneous palette of rare diseases.^1^ Örebro University Hospital services a local population of approximately 300,000 and shares the tertiary specialist care expertise of approximately 2 million people with another hospital in the same region. Thus, there is a limited capacity to recruit a sufficient number of study subjects to analyse swDI’s psychometric properties with modern statistical methods such as factor analysis or item response theory. Engaging more study subjects would certainly substantiate our findings. Although comparable with previous DI validation studies,^4^, ^11^ the lean study sample combined with the heterogeneity of the study subjects’ condition and the lack of a sample size calculation based on a power analysis, is undoubtedly considered as a primary limitation of the present study.

Moreover, a discrepancy could be observed between the subjects involved in the different phases of the validation process as seen in Table 3. A second evaluation with DI before intervention is missing in more than half of the patient subgroup due to the fact that these patients were planned for an intervention by other physicians not participating in the study; hence, the only opportunity to fill in the questionnaire was on the day of intervention. Furthermore, one subject in the patient group missed to grade the experienced discomfort with the VAS scale.

As shown in Table 4, our findings are in conformity with COSMIN criteria for good measurement properties.^14^, ^23^ The lack of any other validated PRO instrument in Swedish, specifically targeting patients with upper airway dyspnoea, did not allow the comparison of the swDI with an existing validated questionnaire. The VAS has been extensively used in the evaluation of health states for many years and in different settings.^15^ These scales are considered to be sensitive, functional and easy to complete,^15^, ^25^, ^26^ yet their psychometric properties are clearly disputed.^15^, ^27^ Thus, the use of a VAS scale in our study, although not a “gold standard,” was an attempt to study swDI’s convergent validity. The VAS scores at exertion moderately correlated with the swDI scores (*r* = .59, ρ = 0.63), consistent with the COSMIN guidelines.^14^ However, VAS scores at rest exhibited weaker correlations with swDI scores (0.42), which was still regarded as moderate,^28^ and can be explained by the fact that the PVFM patients included in our study did not experience any problems breathing at rest.

Voice and respiration are two vital functions sharing the same anatomical subsite in the upper airway: larynx. Dysphonia is present in only 12% of patients with PVFM.^29^ Laryngotracheal stenosis and bilateral vocal fold paralysis generally cause only a mild grade of voice problems as perceived by the patients. However, this is commonly not a concern due to substantially deteriorated breathing.^30^, ^31^ Therefore, we chose the VHI to evaluate the swDI’s discriminant validity considering that the two instruments are intended to measure two different constructs (breathing problems and dysphonia) originating from the same anatomical location. According to Prinsen et al,^14^ instrument correlations measuring related but dissimilar constructs should be lower, that is between 0.3 and 0.5 yet with a minimum of 0.10 from correlations with instruments measuring similar constructs. In our study, VHI scores poorly correlated with swDI scores (*r* = .36, ρ = 0.29), suggesting that the two questionnaires assess two divergent factors.

Given the controversy whether data obtained from Likert scale questionnaires could be considered as interval or ordinal not following a normal distribution,^32^, ^33^, ^34^, ^35^ we purposely analysed our data with both parametrical (eg *t* test, Pearson's *r*, ICC) and non‐parametrical tests (Mann‐Whitney, Wilcoxon Sign, Spearman's ρ). Yet, the results acquired where similar.

An interpretability analysis, as defined by the COSMIN initiative guidelines,^14^, ^23^, ^36^ was missing from the development process of the original DI.^4^ Our study could not provide such data due to the aforementioned limitations concerning the recruitment of a sufficient number of study subjects related to the rarity of the condition. Another limitation was that the smallest detectable change (SDC) and minimal important change (MIC), both measurements defining responsiveness, were also not evaluated. However, the as the basic methodological principles during the validation process were in agreement with the COSMIN guidelines, this study could further be used as a reference for later validation attempts of the DI in other languages.

### 4.4 Clinical applicability of the study

We consider that the swDI is filling the lack of an instrument for Swedish‐speaking patients with upper airway dyspnoea and will certainly be used both in clinical investigations and praxis. As the interest in quality of life is emerging worldwide in both socioeconomics and research, our validation study may act as an inspiration for future studies translating and validating the DI further, thus broaden the use of this robust and easy to use questionnaire globally.

A future study in collaboration with other centres, having access to a larger population would facilitate a further individual analysis of interitem correlations with advanced statistics and consolidate the present validation with traditional statistical methods. It would also allow an anchor‐based longitudinal assessment of at least 4 subgroups within the study subjects, which is needed to analyse SDC and MIC in detail.

The data that support the findings of this study are available on request from the corresponding author. The data are not publicly available due to privacy or ethical restrictions.

## CONFLICT OF INTEREST

None.

## AUTHOR CONTRIBUTIONS

E.N: Conceptualisation (lead), data curation, formal analysis (equal), funding acquisition, investigation (lead), methodology (lead), project administration (supporting), resources, writing (original draft).O.B: Formal analysis (equal), writing (review & editing, supporting). M.vB: Conceptualisation (supporting), investigation (supporting), methodology (supporting), project administration (lead), supervision, writing (review & editing, lead).

## Appendix 1

### Original English version of the Dyspnea Index. (with permission from Jackie Gartner‐Schmidt)

These are some symptoms that you may be feeling. Please circle the response that indicates how frequently you experience the same symptoms: (0 = never, 1 = almost never, 2 = sometimes, 3 = almost always, 4 = always)

**Table:**

1.	I have trouble getting air in		0	1	2	3	4
2.	I feel tightness in my throat when I am having my breathing problem		0	1	2	3	4
3.	It takes more effort to breathe than it used to		0	1	2	3	4
4.	Changes in weather affect my breathing problem		0	1	2	3	4
5.	My breathing gets worse with stress		0	1	2	3	4
6.	I make sound/noise when I breath in		0	1	2	3	4
7.	I have to strain to breathe		0	1	2	3	4
8.	My shortness of breath gets worse with exercise or physical activity		0	1	2	3	4
9.	My breathing problem makes me feel stressed		0	1	2	3	4
10.	My breathing problem causes me to restrict my personal and social life		0	1	2	3	4

## Appendix 2

### Final Swedish version of the Dyspnea Index (SWDI)

Här följer några symptom du kanske känner av. Sätt en ring runt den siffra som bäst motsvarar hur ofta du upplever de olika symptomen (0 = aldrig, 1 = nästan aldrig, 2 = ibland, 3 = nästan alltid, 4 = alltid)

**Table:**

1.	Jag har problem att andas in	0	1	2	3	4
2.	Det känns trångt i halsen när jag har besvär med andningen	0	1	2	3	4
3.	Det är mer ansträngande att andas nu jämfört med tidigare	0	1	2	3	4
4.	När vädret ändras påverkas mina andningsbesvär	0	1	2	3	4
5.	Jag har svårare att andas när jag blir stressad	0	1	2	3	4
6.	Det låter när jag andas in	0	1	2	3	4
7.	Jag måste anstränga mig för att andas	0	1	2	3	4
8.	Mina andningsbesvär blir värre vid träning och fysisk aktivitet	0	1	2	3	4
9.	Mina andningsbesvär gör att jag känner mig stressad	0	1	2	3	4
10.	Mina andningsbesvär begränsar mitt personliga och sociala liv	0	1	2	3	4

## References

[1] Noud M , Hovis K , Gelbard A , et al. Patient‐reported outcome measures in upper airway‐related dyspnea: a systematic review. JAMA Otolaryngol Head Neck Surg. 2017;143(8):824‐831. 28594976 10.1001/jamaoto.2017.0348 PMC5604091

[2] Christopher KL , Morris MJ . Vocal cord dysfunction, paradoxic vocal fold motion, or laryngomalacia? Our understanding requires an interdisciplinary approach. Otolaryngol Clin North Am. 2010;43(1):43‐66, viii. 20172256 10.1016/j.otc.2009.12.002

[3] Snow G , Guardiani E . Movement disorders and voice. Otolaryngol Clin North Am. 2019;52(4):759‐767. 31076164 10.1016/j.otc.2019.03.018

[4] Gartner‐Schmidt JL , Shembel AC , Zullo TG , Rosen CA . Development and validation of the Dyspnea Index (DI): a severity index for upper airway‐related dyspnea. J Voice. 2014;28(6):775‐782. 25311596 10.1016/j.jvoice.2013.12.017

[5] Morris IR . Functional anatomy of the upper airway. Emerg Med Clin North Am. 1988;6(4):639‐669. 3056703

[6] Strohl KP , Butler JP , Malhotra A . Mechanical properties of the upper airway. Compr Physiol. 2012;2(3):1853‐1872. 23723026 10.1002/cphy.c110053 PMC3770742

[7] Pohunek P . Development, structure and function of the upper airways. Paediatr Respir Rev. 2004;5(1):2‐8. 15222948 10.1016/j.prrv.2003.09.002

[8] Schunemann HJ , Griffith L , Jaeschke R , et al. A comparison of the original chronic respiratory questionnaire with a standardized version. Chest. 2003;124(4):1421‐1429. 14555575 10.1378/chest.124.4.1421

[9] Nouraei SA , Randhawa PS , Koury EF , et al. Validation of the Clinical COPD Questionnaire as a psychophysical outcome measure in adult laryngotracheal stenosis. Clin Otolaryngol. 2009;34(4):343‐348. 19673982 10.1111/j.1749-4486.2009.01969.x

[10] Nouraei SA , Nouraei SM , Randhawa PS , et al. Sensitivity and responsiveness of the Medical Research Council dyspnoea scale to the presence and treatment of adult laryngotracheal stenosis. Clin Otolaryngol. 2008;33(6):575‐580. 19126132 10.1111/j.1749-4486.2008.01832.x

[11] De Guzman V , Ballif CL , Maurer R , Hartnick CJ , Raol N . Validation of the dyspnea index in adolescents with exercise‐induced paradoxical vocal fold motion. JAMA Otolaryngol Head Neck Surg. 2014;140(9):823‐828. 25104182 10.1001/jamaoto.2014.1405

[12] Tie K , Buckmire RA , Shah RN . The role of spirometry and dyspnea index in the management of subglottic stenosis. Laryngoscope. 2020;130:2760‐2766. 31603579 10.1002/lary.28337

[13] Ohlsson AC , Dotevall H . Voice handicap index in Swedish. Logoped Phoniatr Vocol. 2009;34(2):60‐66. 19308791 10.1080/14015430902839185

[14] Prinsen CAC , Mokkink LB , Bouter LM , et al. COSMIN guideline for systematic reviews of patient‐reported outcome measures. Qual Life Res. 2018;27(5):1147‐1157. 29435801 10.1007/s11136-018-1798-3 PMC5891568

[15] Fayers PM , Machin D . Quality of Life: The Assessment, Analysis and Reporting of Patient‐reported Outcomes. 2016. West Sussex, UK: Wiley‐Blackwell (an imprint of John Wiley &Sons Ltd).

[16] Epstein J , Santo RM , Guillemin F . A review of guidelines for cross‐cultural adaptation of questionnaires could not bring out a consensus. J Clin Epidemiol. 2015;68(4):435‐441. 25698408 10.1016/j.jclinepi.2014.11.021

[17] Schmidt S , Bullinger M . Current issues in cross‐cultural quality of life instrument development. Arch Phys Med Rehabil. 2003;84:S29‐S34. 12692769 10.1053/apmr.2003.50244

[18] Danielsen AK , Pommergaard HC , Burcharth J , Angenete E , Rosenberg J . Translation of questionnaires measuring health related quality of life is not standardized: a literature based research study. PLoS One. 2015;10(5):e0127050. 25965447 10.1371/journal.pone.0127050 PMC4428794

[19] Swaine‐Verdier A , Doward LC , Hagell P , Thorsen H , McKenna SP . Adapting quality of life instruments. Value Health. 2004;7(Suppl 1):S27‐S30. 15367241 10.1111/j.1524-4733.2004.7s107.x

[20] Wild D , Grove A , Martin M , et al. Principles of good practice for the translation and cultural adaptation process for patient‐reported outcomes (PRO) measures: report of the ISPOR task force for translation and cultural adaptation. Value Health. 2005;8(2):94‐104. 15804318 10.1111/j.1524-4733.2005.04054.x

[21] Kimberlin CL , Winterstein AG . Validity and reliability of measurement instruments used in research. Am J Health‐Syst Pharm. 2008;65(23):2276‐2284. 19020196 10.2146/ajhp070364

[22] Edelen MO , Reeve BB . Applying item response theory (IRT) modeling to questionnaire development, evaluation, and refinement. Qual Life Res. 2007;16(Suppl 1):5‐18. 17375372 10.1007/s11136-007-9198-0

[23] Terwee CB , Bot SD , de Boer MR , et al. Quality criteria were proposed for measurement properties of health status questionnaires. J Clin Epidemiol. 2007;60(1):34‐42. 17161752 10.1016/j.jclinepi.2006.03.012

[24] Cappelleri JC , Jason Lundy J , Hays RD . Overview of classical test theory and item response theory for the quantitative assessment of items in developing patient‐reported outcomes measures. Clin Ther. 2014;36(5):648‐662. 24811753 10.1016/j.clinthera.2014.04.006 PMC4096146

[25] Hjermstad MJ , Fayers PM , Haugen DF , et al. Studies comparing Numerical Rating Scales, Verbal Rating Scales, and Visual Analogue Scales for assessment of pain intensity in adults: a systematic literature review. J Pain Symptom Manage. 2011;41(6):1073‐1093. 21621130 10.1016/j.jpainsymman.2010.08.016

[26] Badia X , Monserrat S , Roset M , Herdman M . Feasibility, validity and test‐retest reliability of scaling methods for health states: the visual analogue scale and the time trade‐off. Qual Life Res. 1999;8(4):303‐310. 10472162 10.1023/a:1008952423122

[27] Chiarotto A , Maxwell LJ , Ostelo RW , Boers M , Tugwell P , Terwee CB . Measurement properties of visual analogue scale, numeric rating scale, and pain severity subscale of the brief pain inventory in patients with low back pain: a systematic review. J Pain. 2019;20(3):245‐263. 30099210 10.1016/j.jpain.2018.07.009

[28] Abma IL , Rovers M , van der Wees PJ . Appraising convergent validity of patient‐reported outcome measures in systematic reviews: constructing hypotheses and interpreting outcomes. BMC Res Notes. 2016;9:226. 27094345 10.1186/s13104-016-2034-2 PMC4837507

[29] Vasudev M . Evaluation of paradoxical vocal fold motion. Ann Allergy Asthma Immunol. 2012;109(4):233‐236. 23010227 10.1016/j.anai.2012.07.006

[30] Nawka T , Sittel C , Arens C , et al. Voice and respiratory outcomes after permanent transoral surgery of bilateral vocal fold paralysis. Laryngoscope. 2015;125(12):2749‐2755. 26235099 10.1002/lary.25415

[31] Hoffman MR , Brand WT , Dailey SH . Effects of balloon dilation for idiopathic laryngotracheal stenosis on voice production. Ann Otol Rhinol Laryngol. 2016;125(1):12‐19. 26180179 10.1177/0003489415595425

[32] Jamieson S . Likert scales: how to (ab)use them. Med Educ. 2004;38(12):1217‐1218. 15566531 10.1111/j.1365-2929.2004.02012.x

[33] Norman G . Likert scales, levels of measurement and the “laws” of statistics. Adv Health Sci Educ. 2010;15(5):625‐632. 10.1007/s10459-010-9222-y 20146096

[34] Carifio J , Perla R . Resolving the 50‐year debate around using and misusing Likert scales. Med Educ. 2008;42(12):1150‐1152. 19120943 10.1111/j.1365-2923.2008.03172.x

[35] Sullivan GM , Artino AR . Analyzing and interpreting data from likert‐type scales. J Grad Med Educ. 2013;5(4):541‐542. 24454995 10.4300/JGME-5-4-18 PMC3886444

[36] Mokkink LB , Terwee CB , Patrick DL , et al. The COSMIN study reached international consensus on taxonomy, terminology, and definitions of measurement properties for health‐related patient‐reported outcomes. J Clin Epidemiol. 2010;63(7):737‐745. 20494804 10.1016/j.jclinepi.2010.02.006

